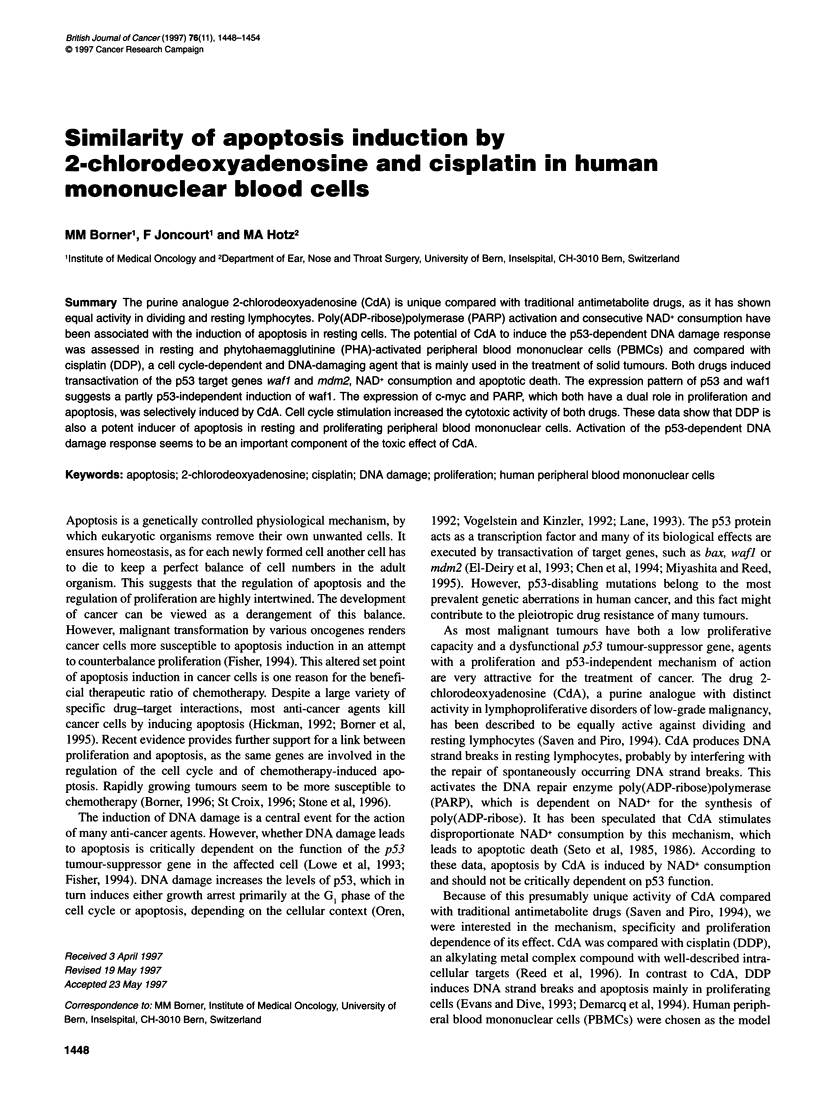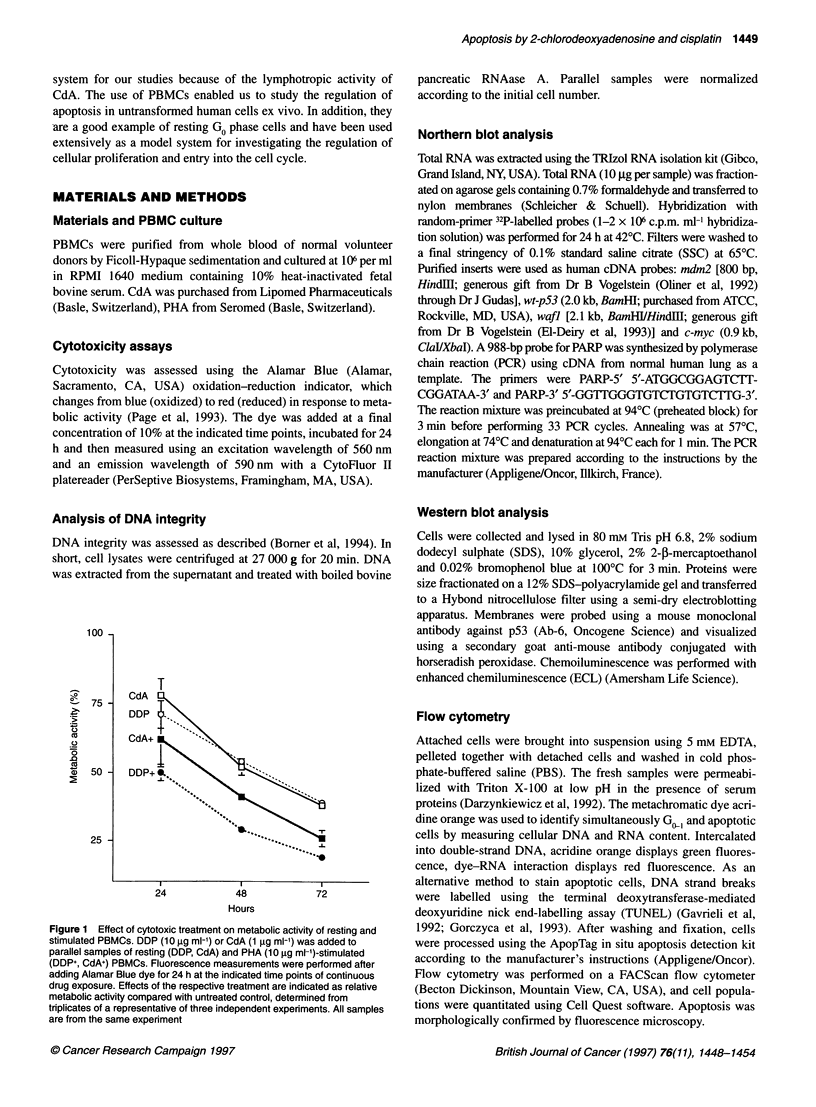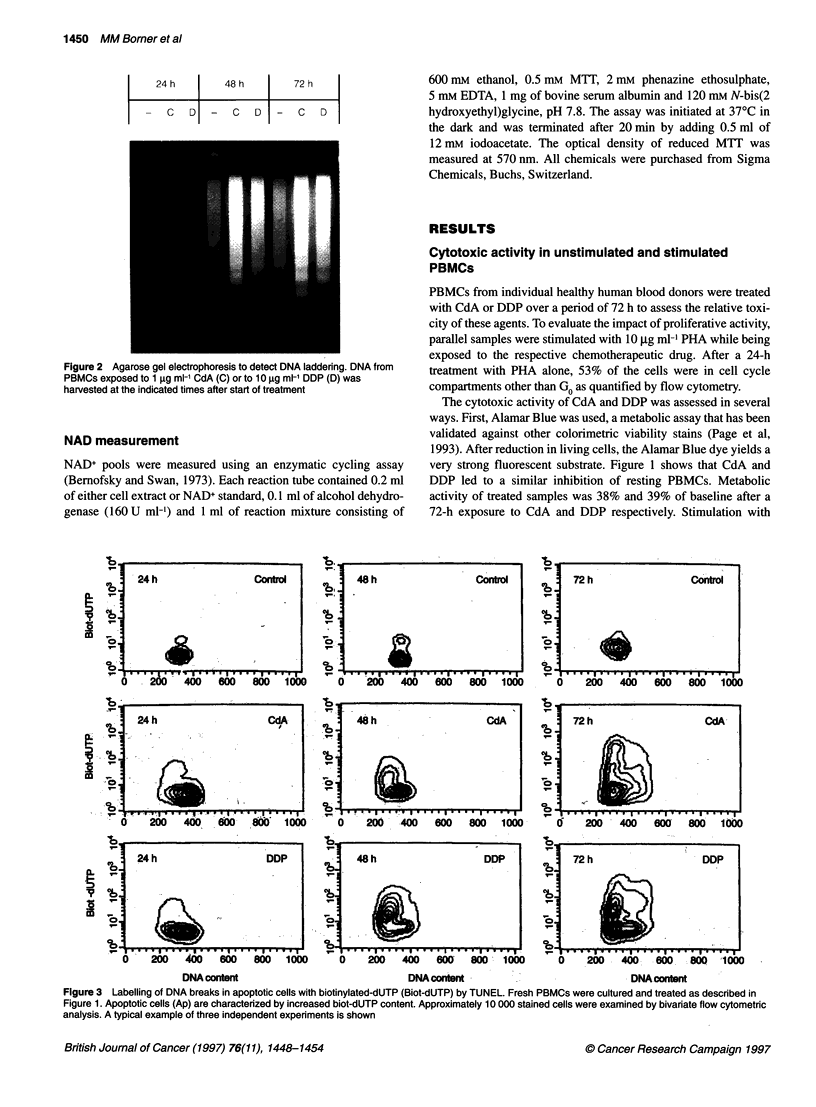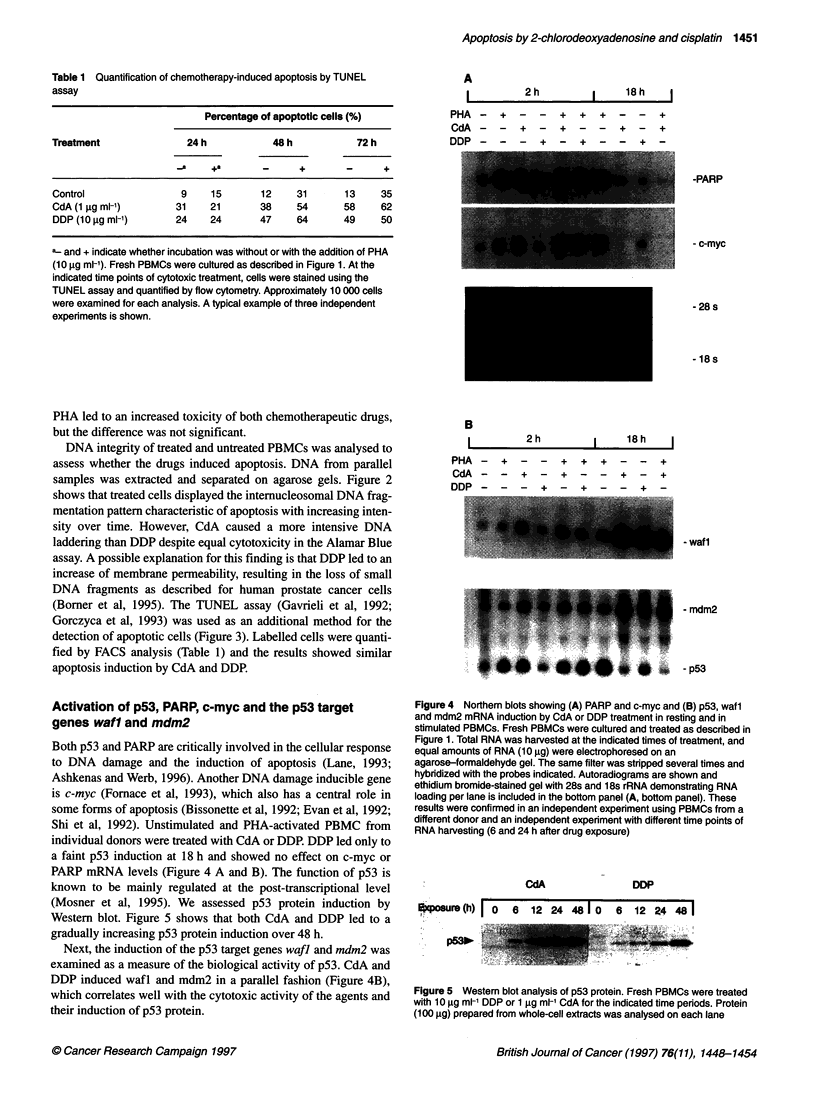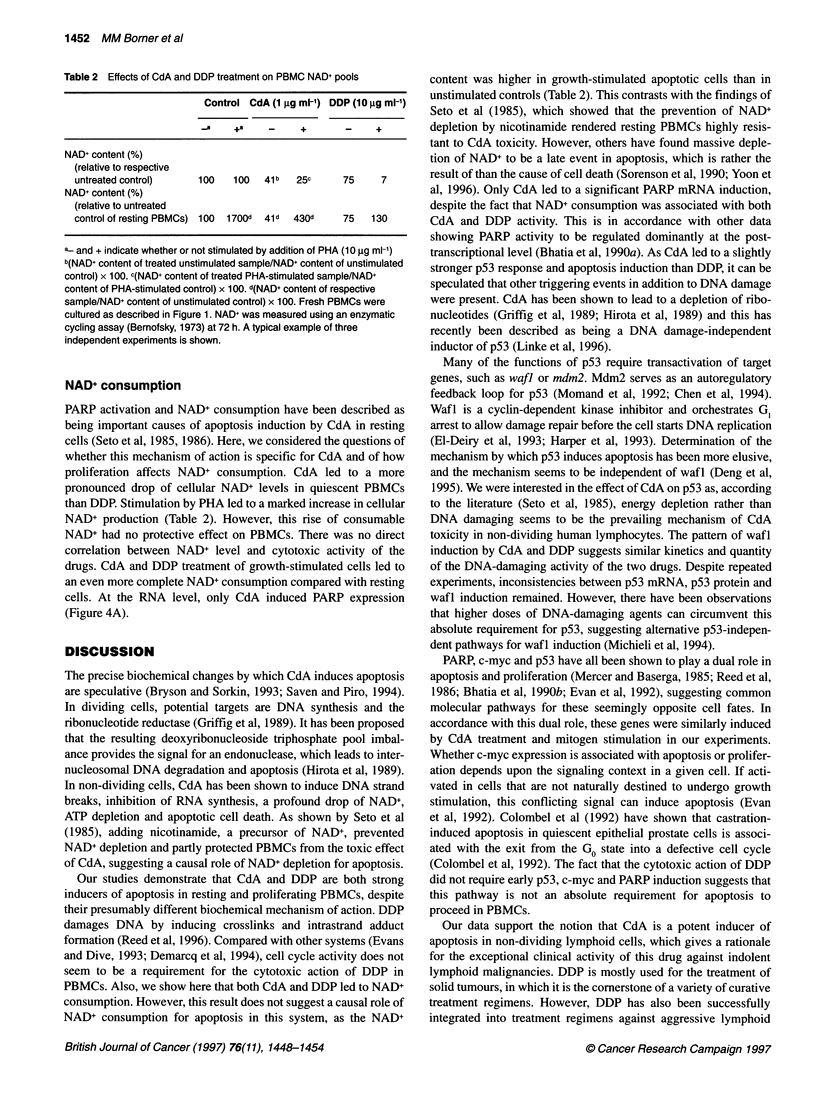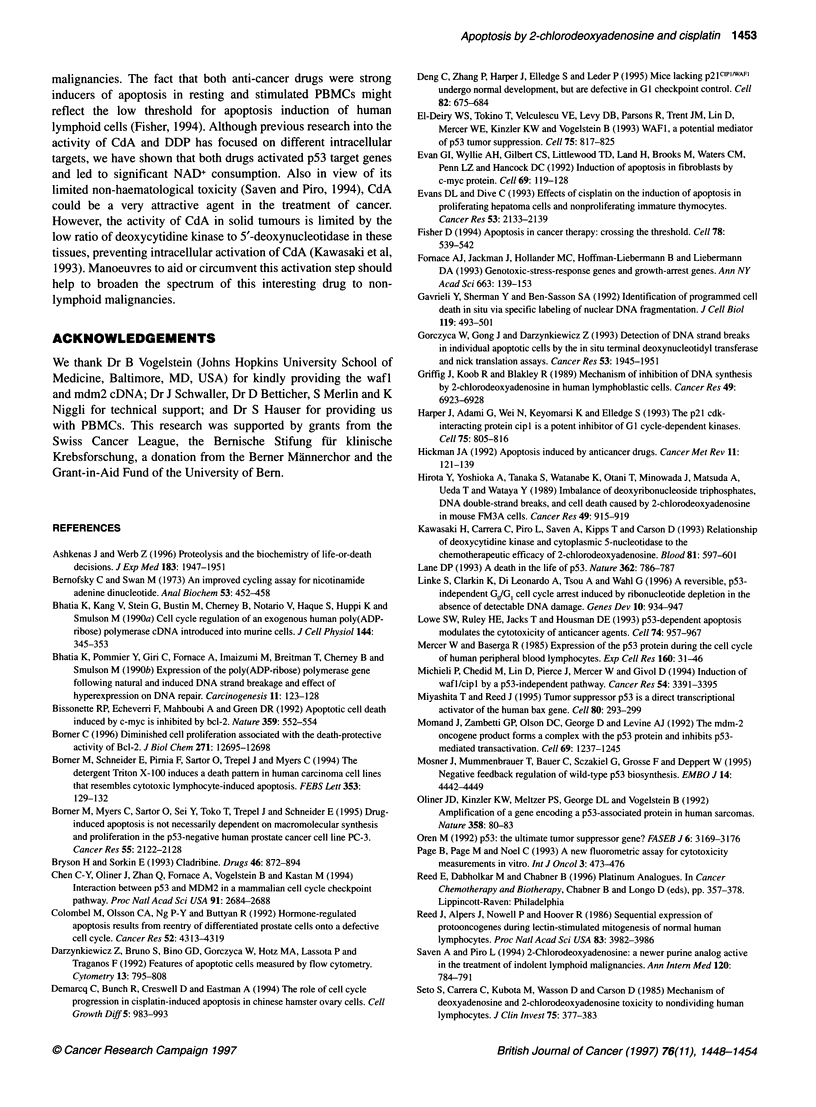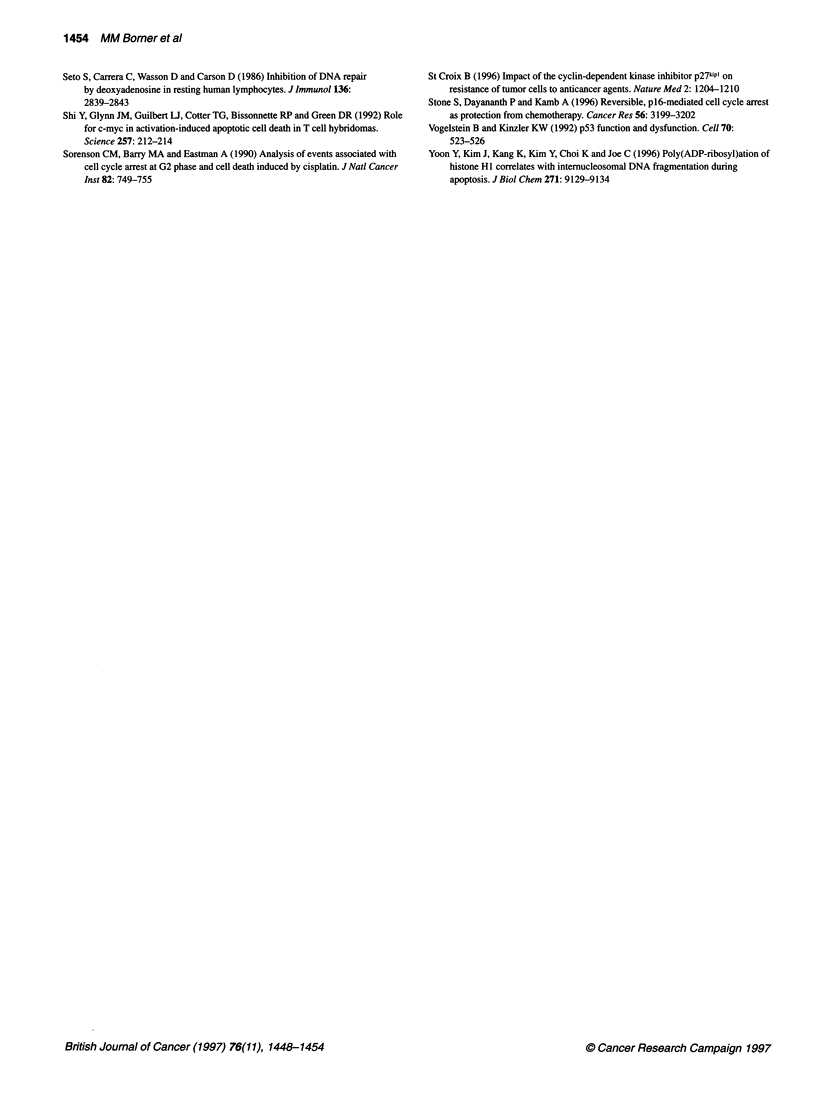# Similarity of apoptosis induction by 2-chlorodeoxyadenosine and cisplatin in human mononuclear blood cells.

**DOI:** 10.1038/bjc.1997.577

**Published:** 1997

**Authors:** M. M. Borner, F. Joncourt, M. A. Hotz

**Affiliations:** Institute of Medical Oncology, University of Bern, Inselspital, Switzerland.

## Abstract

**Images:**


British Journal of Cancer (1997) 76(11), 1448–1454
© 1997 Cancer Research Campaign

### Similarity of apoptosis induction by 2-chlorodeoxyadenosine and cisplatin in human mononuclear blood cells

**MM Borner[1], F Joncourt[1] and MA Hotz[2]**

[1]Institute of Medical Oncology and [2]Department of Ear, Nose and Throat Surgery, University of Bern, Inselspital, CH-3010 Bern, Switzerland

**Summary** The purine analogue 2-chlorodeoxyadenosine (CdA) is unique compared with traditional antimetabolite drugs, as it has shown equal activity in dividing and resting lymphocytes. Poly(ADP-ribose)polymerase (PARP) activation and consecutive $NAD^+$ consumption have been associated with the induction of apoptosis in resting cells. The potential of CdA to induce the p53-dependent DNA damage response was assessed in resting and phytohaemagglutinine (PHA)-activated peripheral blood mononuclear cells (PBMCs) and compared with cisplatin (DDP), a cell cycle-dependent and DNA-damaging agent that is mainly used in the treatment of solid tumours. Both drugs induced transactivation of the p53 target genes *waf1* and *mdm2*, $NAD^+$ consumption and apoptotic death. The expression pattern of p53 and waf1 suggests a partly p53-independent induction of waf1. The expression of c-myc and PARP, which both have a dual role in proliferation and apoptosis, was selectively induced by CdA. Cell cycle stimulation increased the cytotoxic activity of both drugs. These data show that DDP is also a potent inducer of apoptosis in resting and proliferating peripheral blood mononuclear cells. Activation of the p53-dependent DNA damage response seems to be an important component of the toxic effect of CdA.

**Keywords:** apoptosis; 2-chlorodeoxyadenosine; cisplatin; DNA damage; proliferation; human peripheral blood mononuclear cells

Apoptosis is a genetically controlled physiological mechanism, by which eukaryotic organisms remove their own unwanted cells. It ensures homeostasis, as for each newly formed cell another cell has to die to keep a perfect balance of cell numbers in the adult organism. This suggests that the regulation of apoptosis and the regulation of proliferation are highly intertwined. The development of cancer can be viewed as a derangement of this balance. However, malignant transformation by various oncogenes renders cancer cells more susceptible to apoptosis induction in an attempt to counterbalance proliferation (Fisher, 1994). This altered set point of apoptosis induction in cancer cells is one reason for the beneficial therapeutic ratio of chemotherapy. Despite a large variety of specific drug–target interactions, most anti-cancer agents kill cancer cells by inducing apoptosis (Hickman, 1992; Borner et al, 1995). Recent evidence provides further support for a link between proliferation and apoptosis, as the same genes are involved in the regulation of the cell cycle and of chemotherapy-induced apoptosis. Rapidly growing tumours seem to be more susceptible to chemotherapy (Borner, 1996; St Croix, 1996; Stone et al, 1996).

The induction of DNA damage is a central event for the action of many anti-cancer agents. However, whether DNA damage leads to apoptosis is critically dependent on the function of the *p53* tumour-suppressor gene in the affected cell (Lowe et al, 1993; Fisher, 1994). DNA damage increases the levels of p53, which in turn induces either growth arrest primarily at the $G_1$ phase of the cell cycle or apoptosis, depending on the cellular context (Oren, 1992; Vogelstein and Kinzler, 1992; Lane, 1993). The p53 protein acts as a transcription factor and many of its biological effects are executed by transactivation of target genes, such as *bax, waf1* or *mdm2* (El-Deiry et al, 1993; Chen et al, 1994; Miyashita and Reed, 1995). However, p53-disabling mutations belong to the most prevalent genetic aberrations in human cancer, and this fact might contribute to the pleiotropic drug resistance of many tumours.

As most malignant tumours have both a low proliferative capacity and a dysfunctional *p53* tumour-suppressor gene, agents with a proliferation and p53-independent mechanism of action are very attractive for the treatment of cancer. The drug 2-chlorodeoxyadenosine (CdA), a purine analogue with distinct activity in lymphoproliferative disorders of low-grade malignancy, has been described to be equally active against dividing and resting lymphocytes (Saven and Piro, 1994). CdA produces DNA strand breaks in resting lymphocytes, probably by interfering with the repair of spontaneously occurring DNA strand breaks. This activates the DNA repair enzyme poly(ADP-ribose)polymerase (PARP), which is dependent on $NAD^+$ for the synthesis of poly(ADP-ribose). It has been speculated that CdA stimulates disproportionate $NAD^+$ consumption by this mechanism, which leads to apoptotic death (Seto et al, 1985, 1986). According to these data, apoptosis by CdA is induced by $NAD^+$ consumption and should not be critically dependent on p53 function.

Because of this presumably unique activity of CdA compared with traditional antimetabolite drugs (Saven and Piro, 1994), we were interested in the mechanism, specificity and proliferation dependence of its effect. CdA was compared with cisplatin (DDP), an alkylating metal complex compound with well-described intracellular targets (Reed et al, 1996). In contrast to CdA, DDP induces DNA strand breaks and apoptosis mainly in proliferating cells (Evans and Dive, 1993; Demarcq et al, 1994). Human peripheral blood mononuclear cells (PBMCs) were chosen as the model

*Received 3 April 1997*
*Revised 19 May 1997*
*Accepted 23 May 1997*

*Correspondence to:* MM Borner, Institute of Medical Oncology, University of Bern, Inselspital, CH-3010 Bern, Switzerland system for our studies because of the lymphotropic activity of CdA. The use of PBMCs enabled us to study the regulation of apoptosis in untransformed human cells ex vivo. In addition, they are a good example of resting $G_0$ phase cells and have been used extensively as a model system for investigating the regulation of cellular proliferation and entry into the cell cycle.

### MATERIALS AND METHODS

### Materials and PBMC culture

PBMCs were purified from whole blood of normal volunteer donors by Ficoll-Hypaque sedimentation and cultured at $10^6$ per ml in RPMI 1640 medium containing 10% heat-inactivated fetal bovine serum. CdA was purchased from Lipomed Pharmaceuticals (Basle, Switzerland), PHA from Seromed (Basle, Switzerland).

### Cytotoxicity assays

Cytotoxicity was assessed using the Alamar Blue (Alamar, Sacramento, CA, USA) oxidation–reduction indicator, which changes from blue (oxidized) to red (reduced) in response to metabolic activity (Page et al, 1993). The dye was added at a final concentration of 10% at the indicated time points, incubated for 24 h and then measured using an excitation wavelength of 560 nm and an emission wavelength of 590 nm with a CytoFluor II platereader (PerSeptive Biosystems, Framingham, MA, USA).

### Analysis of DNA integrity

DNA integrity was assessed as described (Borner et al, 1994). In short, cell lysates were centrifuged at 27 000 g for 20 min. DNA was extracted from the supernatant and treated with boiled bovine pancreatic RNAase A. Parallel samples were normalized according to the initial cell number.

**Figure 1** Effect of cytotoxic treatment on metabolic activity of resting and stimulated PBMCs. DDP (10 μg ml$^{-1}$) or CdA (1 μg ml$^{-1}$) was added to parallel samples of resting (DDP, CdA) and PHA (10 μg ml$^{-1}$)-stimulated (DDP$^+$, CdA$^+$) PBMCs. Fluorescence measurements were performed after adding Alamar Blue dye for 24 h at the indicated time points of continuous drug exposure. Effects of the respective treatment are indicated as relative metabolic activity compared with untreated control, determined from triplicates of a representative of three independent experiments. All samples are from the same experiment

### Northern blot analysis

Total RNA was extracted using the TRIzol RNA isolation kit (Gibco, Grand Island, NY, USA). Total RNA (10 μg per sample) was fractionated on agarose gels containing 0.7% formaldehyde and transferred to nylon membranes (Schleicher & Schuell). Hybridization with random-primer $^{32}$P-labelled probes (1–2 × $10^6$ c.p.m. ml$^{-1}$ hybridization solution) was performed for 24 h at 42°C. Filters were washed to a final stringency of 0.1% standard saline citrate (SSC) at 65°C. Purified inserts were used as human cDNA probes: *mdm2* [800 bp, *Hin*dIII; generous gift from Dr B Vogelstein (Oliner et al, 1992) through Dr J Gudas], *wt-p53* (2.0 kb, *Bam*HI; purchased from ATCC, Rockville, MD, USA), *waf1* [2.1 kb, *Bam*HI/*Hin*dIII; generous gift from Dr B Vogelstein (El-Deiry et al, 1993)] and *c-myc* (0.9 kb, *Cla*I/*Xba*I). A 988-bp probe for PARP was synthesized by polymerase chain reaction (PCR) using cDNA from normal human lung as a template. The primers were PARP-5′ 5′-ATGGCGGAGTCTT-CGGATAA-3′ and PARP-3′ 5′-GGTTGGGTGTCTGTGTCTTG-3′. The reaction mixture was preincubated at 94°C (preheated block) for 3 min before performing 33 PCR cycles. Annealing was at 57°C, elongation at 74°C and denaturation at 94°C each for 1 min. The PCR reaction mixture was prepared according to the instructions by the manufacturer (Appligene/Oncor, Illkirch, France).

### Western blot analysis

Cells were collected and lysed in 80 mM Tris pH 6.8, 2% sodium dodecyl sulphate (SDS), 10% glycerol, 2% 2-β-mercaptoethanol and 0.02% bromophenol blue at 100°C for 3 min. Proteins were size fractionated on a 12% SDS–polyacrylamide gel and transferred to a Hybond nitrocellulose filter using a semi-dry electroblotting apparatus. Membranes were probed using a mouse monoclonal antibody against p53 (Ab-6, Oncogene Science) and visualized using a secondary goat anti-mouse antibody conjugated with horseradish peroxidase. Chemoiluminescence was performed with enhanced chemiluminescence (ECL) (Amersham Life Science).

### Flow cytometry

Attached cells were brought into suspension using 5 mM EDTA, pelleted together with detached cells and washed in cold phosphate-buffered saline (PBS). The fresh samples were permeabilized with Triton X-100 at low pH in the presence of serum proteins (Darzynkiewicz et al, 1992). The metachromatic dye acridine orange was used to identify simultaneously $G_{0-1}$ and apoptotic cells by measuring cellular DNA and RNA content. Intercalated into double-strand DNA, acridine orange displays green fluorescence, dye–RNA interaction displays red fluorescence. As an alternative method to stain apoptotic cells, DNA strand breaks were labelled using the terminal deoxytransferase-mediated deoxyuridine nick end-labelling assay (TUNEL) (Gavrieli et al, 1992; Gorczyca et al, 1993). After washing and fixation, cells were processed using the ApopTag in situ apoptosis detection kit according to the manufacturer's instructions (Appligene/Oncor). Flow cytometry was performed on a FACScan flow cytometer (Becton Dickinson, Mountain View, CA, USA), and cell populations were quantitated using Cell Quest software. Apoptosis was morphologically confirmed by fluorescence microscopy.

© Cancer Research Campaign 1997

**Figure 2** Agarose gel electrophoresis to detect DNA laddering. DNA from PBMCs exposed to 1 μg ml$^{-1}$ CdA (C) or to 10 μg ml$^{-1}$ DDP (D) was harvested at the indicated times after start of treatment

### NAD measurement

NAD$^+$ pools were measured using an enzymatic cycling assay (Bernofsky and Swan, 1973). Each reaction tube contained 0.2 ml of either cell extract or NAD$^+$ standard, 0.1 ml of alcohol dehydrogenase (160 U ml$^{-1}$) and 1 ml of reaction mixture consisting of 600 mM ethanol, 0.5 mM MTT, 2 mM phenazine ethosulphate, 5 mM EDTA, 1 mg of bovine serum albumin and 120 mM *N*-bis(2 hydroxyethyl)glycine, pH 7.8. The assay was initiated at 37°C in the dark and was terminated after 20 min by adding 0.5 ml of 12 mM iodoacetate. The optical density of reduced MTT was measured at 570 nm. All chemicals were purchased from Sigma Chemicals, Buchs, Switzerland.

### RESULTS

### Cytotoxic activity in unstimulated and stimulated PBMCs

PBMCs from individual healthy human blood donors were treated with CdA or DDP over a period of 72 h to assess the relative toxicity of these agents. To evaluate the impact of proliferative activity, parallel samples were stimulated with 10 μg ml$^{-1}$ PHA while being exposed to the respective chemotherapeutic drug. After a 24-h treatment with PHA alone, 53% of the cells were in cell cycle compartments other than $G_0$ as quantified by flow cytometry.

The cytotoxic activity of CdA and DDP was assessed in several ways. First, Alamar Blue was used, a metabolic assay that has been validated against other colorimetric viability stains (Page et al, 1993). After reduction in living cells, the Alamar Blue dye yields a very strong fluorescent substrate. Figure 1 shows that CdA and DDP led to a similar inhibition of resting PBMCs. Metabolic activity of treated samples was 38% and 39% of baseline after a 72-h exposure to CdA and DDP respectively. Stimulation with

**Figure 3** Labelling of DNA breaks in apoptotic cells with biotinylated-dUTP (Biot-dUTP) by TUNEL. Fresh PBMCs were cultured and treated as described in Figure 1. Apoptotic cells (Ap) are characterized by increased biot-dUTP content. Approximately 10 000 stained cells were examined by bivariate flow cytometric analysis. A typical example of three independent experiments is shown

© Cancer Research Campaign 1997

**Table 1** Quantification of chemotherapy-induced apoptosis by TUNEL assay

| | Percentage of apoptotic cells (%) | | | | | |
|---|---|---|---|---|---|---|
| **Treatment** | **24 h** | | **48 h** | | **72 h** | |
| | –[a] | +[a] | – | + | – | + |
| Control | 9 | 15 | 12 | 31 | 13 | 35 |
| CdA (1 µg ml⁻¹) | 31 | 21 | 38 | 54 | 58 | 62 |
| DDP (10 µg ml⁻¹) | 24 | 24 | 47 | 64 | 49 | 50 |

[a]– and + indicate whether incubation was without or with the addition of PHA (10 µg ml⁻¹). Fresh PBMCs were cultured as described in Figure 1. At the indicated time points of cytotoxic treatment, cells were stained using the TUNEL assay and quantified by flow cytometry. Approximately 10 000 cells were examined for each analysis. A typical example of three independent experiments is shown.

PHA led to an increased toxicity of both chemotherapeutic drugs, but the difference was not significant.

DNA integrity of treated and untreated PBMCs was analysed to assess whether the drugs induced apoptosis. DNA from parallel samples was extracted and separated on agarose gels. Figure 2 shows that treated cells displayed the internucleosomal DNA fragmentation pattern characteristic of apoptosis with increasing intensity over time. However, CdA caused a more intensive DNA laddering than DDP despite equal cytotoxicity in the Alamar Blue assay. A possible explanation for this finding is that DDP led to an increase of membrane permeability, resulting in the loss of small DNA fragments as described for human prostate cancer cells (Borner et al, 1995). The TUNEL assay (Gavrieli et al, 1992; Gorczyca et al, 1993) was used as an additional method for the detection of apoptotic cells (Figure 3). Labelled cells were quantified by FACS analysis (Table 1) and the results showed similar apoptosis induction by CdA and DDP.

### Activation of p53, PARP, c-myc and the p53 target genes *waf1* and *mdm2*

Both p53 and PARP are critically involved in the cellular response to DNA damage and the induction of apoptosis (Lane, 1993; Ashkenas and Werb, 1996). Another DNA damage inducible gene is *c-myc* (Fornace et al, 1993), which also has a central role in some forms of apoptosis (Bissonette et al, 1992; Evan et al, 1992; Shi et al, 1992). Unstimulated and PHA-activated PBMC from individual donors were treated with CdA or DDP. DDP led only to a faint p53 induction at 18 h and showed no effect on c-myc or PARP mRNA levels (Figure 4 A and B). The function of p53 is known to be mainly regulated at the post-transcriptional level (Mosner et al, 1995). We assessed p53 protein induction by Western blot. Figure 5 shows that both CdA and DDP led to a gradually increasing p53 protein induction over 48 h.

Next, the induction of the p53 target genes *waf1* and *mdm2* was examined as a measure of the biological activity of p53. CdA and DDP induced waf1 and mdm2 in a parallel fashion (Figure 4B), which correlates well with the cytotoxic activity of the agents and their induction of p53 protein.

**Figure 4** Northern blots showing (**A**) PARP and c-myc and (**B**) p53, waf1 and mdm2 mRNA induction by CdA or DDP treatment in resting and in stimulated PBMCs. Fresh PBMCs were cultured and treated as described in Figure 1. Total RNA was harvested at the indicated times of treatment, and equal amounts of RNA (10 µg) were electrophoresed on an agarose–formaldehyde gel. The same filter was stripped several times and hybridized with the probes indicated. Autoradiograms are shown and ethidium bromide-stained gel with 28s and 18s rRNA demonstrating RNA loading per lane is included in the bottom panel (**A**, bottom panel). These results were confirmed in an independent experiment using PBMCs from a different donor and an independent experiment with different time points of RNA harvesting (6 and 24 h after drug exposure)

**Figure 5** Western blot analysis of p53 protein. Fresh PBMCs were treated with 10 µg ml⁻¹ DDP or 1 µg ml⁻¹ CdA for the indicated time periods. Protein (100 µg) prepared from whole-cell extracts was analysed on each lane

**Table 2** Effects of CdA and DDP treatment on PBMC NAD+ pools

| | Control | | CdA (1 μg ml−1) | | DDP (10 μg ml−1) | |
|---|---|---|---|---|---|---|
| | –[a] | +[a] | – | + | – | + |
| NAD+ content (%) (relative to respective untreated control) | 100 | 100 | 41[b] | 25[c] | 75 | 7 |
| NAD+ content (%) (relative to untreated control of resting PBMCs) | 100 | 1700[d] | 41[d] | 430[d] | 75 | 130 |

[a]– and + indicate whether or not stimulated by addition of PHA (10 μg ml−1) [b](NAD+ content of treated unstimulated sample/NAD+ content of unstimulated control) × 100. [c](NAD+ content of treated PHA-stimulated sample/NAD+ content of PHA-stimulated control) × 100. [d](NAD+ content of respective sample/NAD+ content of unstimulated control) × 100. Fresh PBMCs were cultured as described in Figure 1. NAD+ was measured using an enzymatic cycling assay (Bernofsky, 1973) at 72 h. A typical example of three independent experiments is shown.

### NAD+ consumption

PARP activation and NAD+ consumption have been described as being important causes of apoptosis induction by CdA in resting cells (Seto et al, 1985, 1986). Here, we considered the questions of whether this mechanism of action is specific for CdA and of how proliferation affects NAD+ consumption. CdA led to a more pronounced drop of cellular NAD+ levels in quiescent PBMCs than DDP. Stimulation by PHA led to a marked increase in cellular NAD+ production (Table 2). However, this rise of consumable NAD+ had no protective effect on PBMCs. There was no direct correlation between NAD+ level and cytotoxic activity of the drugs. CdA and DDP treatment of growth-stimulated cells led to an even more complete NAD+ consumption compared with resting cells. At the RNA level, only CdA induced PARP expression (Figure 4A).

### DISCUSSION

The precise biochemical changes by which CdA induces apoptosis are speculative (Bryson and Sorkin, 1993; Saven and Piro, 1994). In dividing cells, potential targets are DNA synthesis and the ribonucleotide reductase (Griffig et al, 1989). It has been proposed that the resulting deoxyribonucleoside triphosphate pool imbalance provides the signal for an endonuclease, which leads to internucleosomal DNA degradation and apoptosis (Hirota et al, 1989). In non-dividing cells, CdA has been shown to induce DNA strand breaks, inhibition of RNA synthesis, a profound drop of NAD+, ATP depletion and apoptotic cell death. As shown by Seto et al (1985), adding nicotinamide, a precursor of NAD+, prevented NAD+ depletion and partly protected PBMCs from the toxic effect of CdA, suggesting a causal role of NAD+ depletion for apoptosis.

Our studies demonstrate that CdA and DDP are both strong inducers of apoptosis in resting and proliferating PBMCs, despite their presumably different biochemical mechanism of action. DDP damages DNA by inducing crosslinks and intrastrand adduct formation (Reed et al, 1996). Compared with other systems (Evans and Dive, 1993; Demarcq et al, 1994), cell cycle activity does not seem to be a requirement for the cytotoxic action of DDP in PBMCs. Also, we show here that both CdA and DDP led to NAD+ consumption. However, this result does not suggest a causal role of NAD+ consumption for apoptosis in this system, as the NAD+ content was higher in growth-stimulated apoptotic cells than in unstimulated controls (Table 2). This contrasts with the findings of Seto et al (1985), which showed that the prevention of NAD+ depletion by nicotinamide rendered resting PBMCs highly resistant to CdA toxicity. However, others have found massive depletion of NAD+ to be a late event in apoptosis, which is rather the result of than the cause of cell death (Sorenson et al, 1990; Yoon et al, 1996). Only CdA led to a significant PARP mRNA induction, despite the fact that NAD+ consumption was associated with both CdA and DDP activity. This is in accordance with other data showing PARP activity to be regulated dominantly at the post-transcriptional level (Bhatia et al, 1990*a*). As CdA led to a slightly stronger p53 response and apoptosis induction than DDP, it can be speculated that other triggering events in addition to DNA damage were present. CdA has been shown to lead to a depletion of ribonucleotides (Griffig et al, 1989; Hirota et al, 1989) and this has recently been described as being a DNA damage-independent inductor of p53 (Linke et al, 1996).

Many of the functions of p53 require transactivation of target genes, such as *waf1* or *mdm2*. Mdm2 serves as an autoregulatory feedback loop for p53 (Momand et al, 1992; Chen et al, 1994). Waf1 is a cyclin-dependent kinase inhibitor and orchestrates $G_1$ arrest to allow damage repair before the cell starts DNA replication (El-Deiry et al, 1993; Harper et al, 1993). Determination of the mechanism by which p53 induces apoptosis has been more elusive, and the mechanism seems to be independent of waf1 (Deng et al, 1995). We were interested in the effect of CdA on p53 as, according to the literature (Seto et al, 1985), energy depletion rather than DNA damaging seems to be the prevailing mechanism of CdA toxicity in non-dividing human lymphocytes. The pattern of waf1 induction by CdA and DDP suggests similar kinetics and quantity of the DNA-damaging activity of the two drugs. Despite repeated experiments, inconsistencies between p53 mRNA, p53 protein and waf1 induction remained. However, there have been observations that higher doses of DNA-damaging agents can circumvent this absolute requirement for p53, suggesting alternative p53-independent pathways for waf1 induction (Michieli et al, 1994).

PARP, c-myc and p53 have all been shown to play a dual role in apoptosis and proliferation (Mercer and Baserga, 1985; Reed et al, 1986; Bhatia et al, 1990*b*; Evan et al, 1992), suggesting common molecular pathways for these seemingly opposite cell fates. In accordance with this dual role, these genes were similarly induced by CdA treatment and mitogen stimulation in our experiments. Whether c-myc expression is associated with apoptosis or proliferation depends upon the signaling context in a given cell. If activated in cells that are not naturally destined to undergo growth stimulation, this conflicting signal can induce apoptosis (Evan et al, 1992). Colombel et al (1992) have shown that castration-induced apoptosis in quiescent epithelial prostate cells is associated with the exit from the $G_0$ state into a defective cell cycle (Colombel et al, 1992). The fact that the cytotoxic action of DDP did not require early p53, c-myc and PARP induction suggests that this pathway is not an absolute requirement for apoptosis to proceed in PBMCs.

Our data support the notion that CdA is a potent inducer of apoptosis in non-dividing lymphoid cells, which gives a rationale for the exceptional clinical activity of this drug against indolent lymphoid malignancies. DDP is mostly used for the treatment of solid tumours, in which it is the cornerstone of a variety of curative treatment regimens. However, DDP has also been successfully integrated into treatment regimens against aggressive lymphoid

© Cancer Research Campaign 1997

malignancies. The fact that both anti-cancer drugs were strong inducers of apoptosis in resting and stimulated PBMCs might reflect the low threshold for apoptosis induction of human lymphoid cells (Fisher, 1994). Although previous research into the activity of CdA and DDP has focused on different intracellular targets, we have shown that both drugs activated p53 target genes and led to significant $NAD^+$ consumption. Also in view of its limited non-haematological toxicity (Saven and Piro, 1994), CdA could be a very attractive agent in the treatment of cancer. However, the activity of CdA in solid tumours is limited by the low ratio of deoxycytidine kinase to 5′-deoxynucleotidase in these tissues, preventing intracellular activation of CdA (Kawasaki et al, 1993). Manoeuvres to aid or circumvent this activation step should help to broaden the spectrum of this interesting drug to non-lymphoid malignancies.

### ACKNOWLEDGEMENTS

We thank Dr B Vogelstein (Johns Hopkins University School of Medicine, Baltimore, MD, USA) for kindly providing the waf1 and mdm2 cDNA; Dr J Schwaller, Dr D Betticher, S Merlin and K Niggli for technical support; and Dr S Hauser for providing us with PBMCs. This research was supported by grants from the Swiss Cancer League, the Bernische Stifung für klinische Krebsforschung, a donation from the Berner Männerchor and the Grant-in-Aid Fund of the University of Bern.

### REFERENCES

Ashkenas J and Werb Z (1996) Proteolysis and the biochemistry of life-or-death decisions. *J Exp Med* **183**: 1947–1951

Bernofsky C and Swan M (1973) An improved cycling assay for nicotinamide adenine dinucleotide. *Anal Biochem* **53**: 452–458

Bhatia K, Kang V, Stein G, Bustin M, Cherney B, Notario V, Haque S, Huppi K and Smulson M (1990*a*) Cell cycle regulation of an exogenous human poly(ADP-ribose) polymerase cDNA introduced into murine cells. *J Cell Physiol* **144**: 345–353

Bhatia K, Pommier Y, Giri C, Fornace A, Imaizumi M, Breitman T, Cherney B and Smulson M (1990*b*) Expression of the poly(ADP-ribose) polymerase gene following natural and induced DNA strand breakage and effect of hyperexpression on DNA repair. *Carcinogenesis* **11**: 123–128

Bissonette RP, Echeverri F, Mahboubi A and Green DR (1992) Apoptotic cell death induced by c-myc is inhibited by bcl-2. *Nature* **359**: 552–554

Borner C (1996) Diminished cell proliferation associated with the death-protective activity of Bcl-2. *J Biol Chem* **271**: 12695–12698

Borner M, Schneider E, Pirnia F, Sartor O, Trepel J and Myers C (1994) The detergent Triton X-100 induces a death pattern in human carcinoma cell lines that resembles cytotoxic lymphocyte-induced apoptosis. *FEBS Lett* **353**: 129–132

Borner M, Myers C, Sartor O, Sei Y, Toko T, Trepel J and Schneider E (1995) Drug-induced apoptosis is not necessarily dependent on macromolecular synthesis and proliferation in the p53-negative human prostate cancer cell line PC-3. *Cancer Res* **55**: 2122–2128

Bryson H and Sorkin E (1993) Cladribine. *Drugs* **46**: 872–894

Chen C-Y, Oliner J, Zhan Q, Fornace A, Vogelstein B and Kastan M (1994) Interaction between p53 and MDM2 in a mammalian cell cycle checkpoint pathway. *Proc Natl Acad Sci USA* **91**: 2684–2688

Colombel M, Olsson CA, Ng P-Y and Buttyan R (1992) Hormone-regulated apoptosis results from reentry of differentiated prostate cells onto a defective cell cycle. *Cancer Res* **52**: 4313–4319

Darzynkiewicz Z, Bruno S, Bino GD, Gorczyca W, Hotz MA, Lassota P and Traganos F (1992) Features of apoptotic cells measured by flow cytometry. *Cytometry* **13**: 795–808

Demarcq C, Bunch R, Creswell D and Eastman A (1994) The role of cell cycle progression in cisplatin-induced apoptosis in chinese hamster ovary cells. *Cell Growth Diff* **5**: 983–993

Deng C, Zhang P, Harper J, Elledge S and Leder P (1995) Mice lacking $p21^{CIP1/WAF1}$ undergo normal development, but are defective in G1 checkpoint control. *Cell* **82**: 675–684

El-Deiry WS, Tokino T, Velculescu VE, Levy DB, Parsons R, Trent JM, Lin D, Mercer WE, Kinzler KW and Vogelstein B (1993) WAF1, a potential mediator of p53 tumor suppression. *Cell* **75**: 817–825

Evan GI, Wyllie AH, Gilbert CS, Littlewood TD, Land H, Brooks M, Waters CM, Penn LZ and Hancock DC (1992) Induction of apoptosis in fibroblasts by c-myc protein. *Cell* **69**: 119–128

Evans DL and Dive C (1993) Effects of cisplatin on the induction of apoptosis in proliferating hepatoma cells and nonproliferating immature thymocytes. *Cancer Res* **53**: 2133–2139

Fisher D (1994) Apoptosis in cancer therapy: crossing the threshold. *Cell* **78**: 539–542

Fornace AJ, Jackman J, Hollander MC, Hoffman-Liebermann B and Liebermann DA (1993) Genotoxic-stress-response genes and growth-arrest genes. *Ann NY Acad Sci* 663: 139–153

Gavrieli Y, Sherman Y and Ben-Sasson SA (1992) Identification of programmed cell death in situ via specific labeling of nuclear DNA fragmentation. *J Cell Biol* **119**: 493–501

Gorczyca W, Gong J and Darzynkiewicz Z (1993) Detection of DNA strand breaks in individual apoptotic cells by the in situ terminal deoxynucleotidyl transferase and nick translation assays. *Cancer Res* **53**: 1945–1951

Griffig J, Koob R and Blakley R (1989) Mechanism of inhibition of DNA synthesis by 2-chlorodeoxyadenosine in human lymphoblastic cells. *Cancer Res* **49**: 6923–6928

Harper J, Adami G, Wei N, Keyomarsi K and Elledge S (1993) The p21 cdk-interacting protein cip1 is a potent inhibitor of G1 cycle-dependent kinases. *Cell* **75**: 805–816

Hickman JA (1992) Apoptosis induced by anticancer drugs. *Cancer Met Rev* **11**: 121–139

Hirota Y, Yoshioka A, Tanaka S, Watanabe K, Otani T, Minowada J, Matsuda A, Ueda T and Wataya Y (1989) Imbalance of deoxyribonucleoside triphosphates, DNA double-strand breaks, and cell death caused by 2-chlorodeoxyadenosine in mouse FM3A cells. *Cancer Res* **49**: 915–919

Kawasaki H, Carrera C, Piro L, Saven A, Kipps T and Carson D (1993) Relationship of deoxycytidine kinase and cytoplasmic 5-nucleotidase to the chemotherapeutic efficacy of 2-chlorodeoxyadenosine. *Blood* **81**: 597–601

Lane DP (1993) A death in the life of p53. *Nature* **362**: 786–787

Linke S, Clarkin K, Di Leonardo A, Tsou A and Wahl G (1996) A reversible, p53-independent $G_0/G_1$ cell cycle arrest induced by ribonucleotide depletion in the absence of detectable DNA damage. *Genes Dev* **10**: 934–947

Lowe SW, Ruley HE, Jacks T and Housman DE (1993) p53-dependent apoptosis modulates the cytotoxicity of anticancer agents. *Cell* **74**: 957–967

Mercer W and Baserga R (1985) Expression of the p53 protein during the cell cycle of human peripheral blood lymphocytes. *Exp Cell Res* **160**: 31–46

Michieli P, Chedid M, Lin D, Pierce J, Mercer W and Givol D (1994) Induction of waf1/cip1 by a p53-independent pathway. *Cancer Res* **54**: 3391–3395

Miyashita T and Reed J (1995) Tumor suppressor p53 is a direct transcriptional activator of the human bax gene. *Cell* **80**: 293–299

Momand J, Zambetti GP, Olson DC, George D and Levine AJ (1992) The mdm-2 oncogene product forms a complex with the p53 protein and inhibits p53-mediated transactivation. *Cell* **69**: 1237–1245

Mosner J, Mummenbrauer T, Bauer C, Sczakiel G, Grosse F and Deppert W (1995) Negative feedback regulation of wild-type p53 biosynthesis. *EMBO J* **14**: 4442–4449

Oliner JD, Kinzler KW, Meltzer PS, George DL and Vogelstein B (1992) Amplification of a gene encoding a p53-associated protein in human sarcomas. *Nature* **358**: 80–83

Oren M (1992) p53: the ultimate tumor suppressor gene? *FASEB J* **6**: 3169–3176

Page B, Page M and Noel C (1993) A new fluorometric assay for cytotoxicity measurements in vitro. *Int J Oncol* **3**: 473–476

Reed E, Dabholkar M and Chabner B (1996) Platinum Analogues. In *Cancer Chemotherapy and Biotherapy*, Chabner B and Longo D (eds), pp. 357–378. Lippincott-Raven: Philadelphia

Reed J, Alpers J, Nowell P and Hoover R (1986) Sequential expression of protooncogenes during lectin-stimulated mitogenesis of normal human lymphocytes. *Proc Natl Acad Sci USA* **83**: 3982–3986

Saven A and Piro L (1994) 2-Chlorodeoxyadenosine: a newer purine analog active in the treatment of indolent lymphoid malignancies. *Ann Intern Med* **120**: 784–791

Seto S, Carrera C, Kubota M, Wasson D and Carson D (1985) Mechanism of deoxyadenosine and 2-chlorodeoxyadenosine toxicity to nondividing human lymphocytes. *J Clin Invest* **75**: 377–383

© Cancer Research Campaign 1997

Seto S, Carrera C, Wasson D and Carson D (1986) Inhibition of DNA repair by deoxyadenosine in resting human lymphocytes. *J Immunol* **136**: 2839–2843

Shi Y, Glynn JM, Guilbert LJ, Cotter TG, Bissonnette RP and Green DR (1992) Role for c-myc in activation-induced apoptotic cell death in T cell hybridomas. *Science* **257**: 212–214

Sorenson CM, Barry MA and Eastman A (1990) Analysis of events associated with cell cycle arrest at G2 phase and cell death induced by cisplatin. *J Natl Cancer Inst* **82**: 749–755

St Croix B (1996) Impact of the cyclin-dependent kinase inhibitor $p27^{kip1}$ on resistance of tumor cells to anticancer agents. *Nature Med* 2: 1204–1210

Stone S, Dayananth P and Kamb A (1996) Reversible, p16-mediated cell cycle arrest as protection from chemotherapy. *Cancer Res* **56**: 3199–3202

Vogelstein B and Kinzler KW (1992) p53 function and dysfunction. *Cell* **70**: 523–526

Yoon Y, Kim J, Kang K, Kim Y, Choi K and Joe C (1996) Poly(ADP-ribosyl)ation of histone H1 correlates with internucleosomal DNA fragmentation during apoptosis. *J Biol Chem* **271**: 9129–9134

© Cancer Research Campaign 1997